# Pressure–Flow Relation of Porcine Thoracic Duct Segment

**DOI:** 10.3390/bioengineering12040401

**Published:** 2025-04-09

**Authors:** Bhavesh Patel, Xiao Lu, Aashish Ahuja, Jillian N. Noblet, Joshua F. Krieger, Sean Chambers, Max Itkin, Ghassan S. Kassab

**Affiliations:** 1 California Medical Innovations Institute, San Diego, CA 92121, USA; 2 Cook Medical, Bloomington, IN 47402, USA; 3 Center for Lymphatic Disorders, University of Pennsylvania Medical Center, Philadelphia, PA 19104, USA

**Keywords:** flow resistance, mathematical model, lymphatic valve, Poiseuille’s law

## Abstract

The lymphatic system collects excess fluid and molecules from the interstitial space back into the venous system mainly via the thoracic duct (TD). Despite the importance of the lymphatic flow in health and disease, a validated pressure–flow relation of lymphatic fluid through the TD is lacking in a translational large animal model. The objective of this work was to establish a pressure–flow relationship for a TD segment with one valve in a swine model. Our methodology consisted of using a specialized bench experimental setup to measure the passive forward flow of 0.9% saline through single-valve TD segments (*n* = 5) under various pressure gradients. Using Poiseuille’s law, we computed the resistance to flow in the TD segment. Subsequently, we used a sigmoidal function to model the relation between valve resistance and pressure gradient across the valve. We estimated the model’s parameters using the Poiseuille-based resistance values and associated experimental pressure date then validated the model by comparing model predictions of flow to experimental measurements. We found that the model performs accurately ($R^{2}=0.985\pm 0.010$). The resistance model validated here for a single valve TD segment provides a fundamental element for computational simulations of lymphatic flow in the entire TD. Moreover, this work provides a baseline for future characterization of TD behavior under pathological conditions, such as congestive heart failure or hepatitis caused ascites.

## 1. Introduction

The lymphatic system is an extensive network of distensible channels that drain into the veins [1,2,3]. This system collects and transports water, electrolytes, proteins, lipids, and other large molecules from the interstitial space back into the venous system via multiple lympho-venous connections, of which the thoracic duct (TD) is the largest and empties in the jugular and subclavian veins. As the primary collecting vessel in the lymphatic system [4], the TD has attracted particular attention for the treatment of lymphatic as well as venous disorders. There are three main reasons for intervention in the lymphatic system via TD access: (1) decompression of lymphatic system, (2) elimination of toxic substances dissolved in lymph, and (3) depletion of cells circulating in the TD. The decompression drainage, which is commonly used, provides resolution or reduction of the following: venous pressure, distension of veins, peripheral edema, ascites, hepatomegaly, etc. [5,6].

Although drainage has shown efficacy, it is only able to provide temporary relief, and the procedure must be repeated frequently. To develop improved therapies for decompression of the TD, we must understand the pressure–flow relation of the system in health and disease. Indeed, the changes in flow and pressure are affected by lymphatic disorders [7,8]. TD undergoes active and passive driving forces, which return lymph to the venous system. The active driving mechanism is due to the intrinsic contractility of the lymph vessels [9,10,11], while passive forces are applied from extrinsic activities such as respiration [12,13] and arterial pulsation [14,15].

Mathematical models of lymph flow are essential to understanding lymphatic functions in health and disease, as well as in silico design of therapeutic approaches. Lumped models have commonly been used to model lymphatic flow [16,17,18,19,20,21,22]. They are simplified representations of the lymphatic system that use ordinary differential equations (ODEs) [23,24]. Lumped models often use analogies to electric circuits for representing lymphatic vessels and valves as circuit elements. Despite their simplifications, lumped models are very insightful since they can be used to model large networks of lymphatic vessels by minimizing mathematical complications [23,24]. Such models typically require the following mathematical relations for each lymphatic vessel considered in the system: (1) relation between resistance to flow and pressure gradient under passive conditions; (2) relation between resistance to flow and pressure gradient under active conditions; (3) passive pressure–diameter relation of the vessel wall; (4) active pressure–diameter relation of the vessel wall. To our knowledge, only two previous studies have evaluated the resistance to flow of the TD. Both assumed a constant resistance value although it is bound to change due to changes in vessel diameter and valve opening with variation of pressure gradient [25,26].

Accordingly, the objective of this work was to validate a passive pressure–flow relation in swine TD and establish a model of the resistance to flow of the TD segment with one valve. Swine TD was selected given the similarities in the anatomy and physiology between porcine and humans. This validated relation is important because it provides a foundation for developing computational models of lymphatic flow in a translational large animal model. These models, in turn, will allow us to better understand the behavior of the TD vessel and valves under normal conditions which will serve as a basis for understanding lymphatic dysfunction. These models can also help with the virtual testing of therapeutic approaches.

## 2. Materials and Methods

### 2.1. Experimental Setup

**Figure 1:**
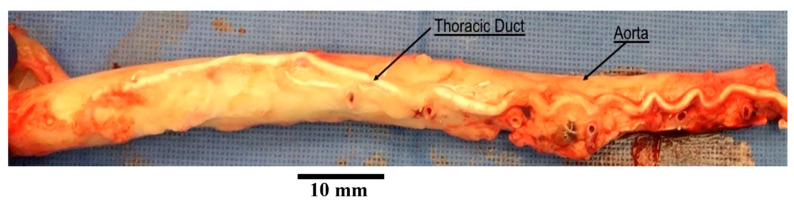
An aorta with thoracic duct harvested from a swine. The thoracic duct shown in this picture was injected with white polymer for the purpose of another study.

**Figure 2:**
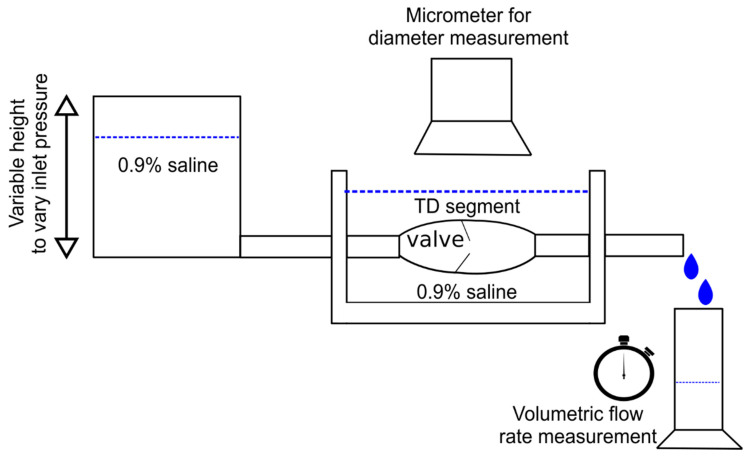
Illustration of the bench setup used the measure change in outer diameter and volumetric flow rate through the TD segment for an imposed pressure gradient.

TD was carefully dissected and harvested from a total of n=5 swine (Yorkshire, either gender, 55 ± 7 kg). All animal experiments were performed in accordance with national and local ethical guidelines, including the Principles of Laboratory Animal Care, the Guide for the Care and Use of Laboratory Animals, and the National Society for Medical Research. The research protocol was approved by California Medical Innovations Institute IACUC. We refer to the “Institutional Review Board Statement” for details about the study permission. We used swine as the experimental model because of its anatomical and physiological similarity to humans [27,28]. The TD was found to be in close vicinity of the aorta, as shown in Figure 1. After the TD was harvested from the swine, a TD segment about 31 mm long was isolated from the mid region of the entire TD (centrally located and equidistant from cisterna chyli and lympho-venous anastomosis) and any identified branch was ligated to prevent leakage during the test. The choice of this length was motivated by our study of the TD structure in swine where it was found that valves were separated on average by 31 mm based on ex vivo measurements. In the same study, it was found that the ratio of the delta between in situ length and ex vivo length divided by the in situ length of the TD was about 30%. The segment was thus mounted on the bench setup, illustrated in Figure 2, stretched by 30% such that its mounted length is L = 40.3 mm. The segment was cannulated at both ends and the entire segment was immersed in a 0.9% saline bath maintained in a temperature-controlled incubator at 37 °C. One end of the canula (referred to as the outlet) was connected to an open tube positioned above a cylindrical container. The other end (referred to as the inlet) was connected to a reservoir filled with 0.9% saline solution. The reservoir was maintained at different heights to generate pressure differences $\Delta p_{total}\;$between 0 and 10 cmH_2_O in increment of 1 cmH_2_O across the setup and flow the saline solution through the segment. This pressure range was selected because our previous study shows that the in vivo pressure gradient from proximal to distal TD was approximately 10 cmH_2_O and the pressure at distal TD was 9 cmH_2_O mmHg [29]. We also found that the TD diameter changed little in diameter vs. pressure relation when pressure was higher than 8 cmH_2_O [30]. Saline solution (μ=0.7 cP, P = poise) was chosen for its very close viscosity to lymph (1 cP [17]). It has been demonstrated in at previous study that the difference in viscosity between saline and lymph does not affect pressure–flow measurements [31]. For each pressure gradient applied, once a steady flow was observed, the outer diameter $D_{out}$ of the TD segment was recorded using a CCD camera mounted on a stereo microscope and measured towards the middle of the segment using Image J v1.52, an open-source and free software for processing and analyzing scientific images [32]. The resulting volumetric flow rate Q was estimated by measuring the volume of saline pouring in the cylindrical container over one minute on the outlet. After the testing was completed, the TD segment was cryo-sectioned transversally, stained by Toluidine blue, and imaged using a histological microscope. The thickness t of the segment was then measured using the software Image J. Subsequently, the segment was cut radially and opened to verify that one (an only one) valve was indeed present. If not, the data were not considered, and the test was repeated with another segment from the same TD until a segment with only one valve was obtained. At most, two repetitions were necessary for each TD to target a segment with one valve.

### 2.2. Pressure, Flow, and Resistance

The pressure gradient across the setup can be expressed as a sum of change in pressure through the elements of the setup (c.f. Figure 2) such that:

(1) $$\Delta p_{total}=\Delta p_{conn}+\Delta p_{vessel}+\Delta p_{valve}$$

Here, $\Delta p_{conn}$ refers to the pressure gradient in the connectors and tubing, $\Delta p_{vessel}$ indicates the pressure gradient along the TD vessel, and $\Delta p_{valve}$ represents the pressure gradient across the valve. Considering the setup to be non-permeable, we assumed that the volumetric flow rate Q was constant across all elements of the setup. Using a lumped model, we assumed that the experimental setup can be represented as an analogous electrical circuit with each element acting as a resistor (i.e., resistance to flow) [17,33,34,35,36]. These assumptions led to the following relation between the total pressure gradient across the setup and the volumetric flow rate:

(2) $$\Delta p_{total}=\left(R_{conn}+R_{vessel}+R_{valve}\right)Q=R_{total}Q$$

Here, $R_{i}$ represents the resistance to flow of the element i of the setup. Specifically, $R_{conn}$ represents the resistance due to the connectors included in the experimental setup, $R_{vessel}$ the resistance due to the vessel wall, and $R_{valve}$ the resistance due to the valve.

#### 2.2.1. Total Resistance of the Setup

The resistance $R_{total}$ across the setup can be calculated directly from the experimental measurements as:

(3) $$R_{total}=\frac{\Delta p_{total}}{Q}$$

#### 2.2.2. Resistance of the Connectors

Since all of the elements connecting the TD segment to the rest of the setup were rigid, the overall resistance of the connectors $R_{conn}$ was assumed to be constant, i.e., independent of pressure and flow conditions as well as independent of the specimen tested. We thus tested a rigid silicon phantom tube (inner diameter $D_{phantom}$ = 3 mm, length $L_{phantom}$ = 31 mm), for which the resistance $R_{phantom}$ could be directly estimated from Poiseuille’s law since the inner diameter and length are constant:

(4) $$R_{phantom}=\frac{128\mu L_{phantom}}{\pi D_{phantom}^{4}}$$

Poiseuille’s law is valid since a Newtonian fluid, 0.9% saline, with very low viscosity was used to flow through the tube where the length of the tube was more than five times its inner diameter. As a result, the flow through the vessel was considered laminar (Reynolds’ number, *Re* < 50) and fully developed ($L_{phantom}$ > 5 × $D_{phantom}$) [37]. For different total pressure gradients applied across the setup, the flow rate of 0.9% saline was measured. Since all the elements were rigid, the pressure–flow relation was assumed linear, and its slope was equal to the total resistance to flow of this setup. The value $R_{phantom}$ was then subtracted from the total resistance of the setup to determine the value of $R_{conn}.$

#### 2.2.3. Resistance of the TD Segment

The resistance to flow of the TD segment is due to the vessel resistance and the valve resistance. The resistance due to vessel can be calculated using Poiseuille’s relation. Assuming that the inner diameter D of the tested segment is constant along its length (for a given overall pressure gradient) the following expressions were given by Poiseuille’s law for the vessel resistance:

(5) $$R_{vessel}=\frac{128\mu L}{\pi D^{4}}$$

The inner diameter D was calculated for each segment using the outer diameter measured during the experiment and assuming that the thickness did not change significantly from the unloaded state such that $D=D_{out}-t$, where t designates the thickness. The resistance to flow of the valve was then calculated from Equation (2) as:

(6) $$R_{valve}=R_{total}-(R_{conn}+R_{vessel})$$

### 2.3. Model of the Valve Resistance

While a model of the vessel resistance was directly given by Poiseuille’s law, it was necessary to establish a mathematical relation for the valve resistance. It has been shown in previous studies that the lymphatic valve resistance to forward flow can be modeled by the following sigmoidal relation [17,33,34]:

(7) $$R_{valve}^{m}=R_{vl}+R_{vh}\left(\frac{1}{1+e^{s∆p_{valve}}}\right)$$

$∆p_{valve}$ designates the pressure gradient across the valve. The parameter $R_{vl}$ (>0) represents the low resistance of the valve at high pressure gradient across the valve (i.e., when the valve reaches maximum opening) while $R_{vl}+R_{vh}$ (>0) represents the maximum resistance of the valve (achieved for large negative pressure gradients). The parameter s (>0) refers to the slope of the transition from high to low resistance values as pressure gradient increases. We applied this model for the TD valve. First, the pressure gradient across the valve was obtained from experimental data as:

(8) $${∆p}_{valve}={∆p}_{total}-(R_{conn}+R_{vessel})Q$$

To ensure consistent values from a physics standpoint, an exponential model was fitted to the behavior of ${∆p}_{valve}$ as a function of ${∆p}_{total}$ such that:

(9) $$∆p_{valve}^{m}=P_{0}e^{A{∆p}_{total}}$$

where the superscript m indicates model based values, and $P_{0}$ (>0) and A (no bounds) designate a model parameter. Using resulting values $∆p_{valve}^{m}$ from the fitting and the value of $R_{valve}$ obtained with Equation (6) based on the experimental data, the model parameters $R_{vl}$, $R_{vh}$, and s were established through model fitting. The model was validated by comparing experimentally measured flow Q for each TD segment to flow $Q^{m}$ estimated based on valve resistance model:

(10) $$Q^{m}=\frac{{∆p}_{total}}{R_{conn}+R_{vessel}+R_{valve}^{m}}$$

In the model, $R_{vl}$ represents the lowest valve resistance value attained at very high pressure across the TD valve. We estimated the threshold value $∆p_{valve}^{t}$ at which the valve resistance is within 5% of $R_{vl}$. This value represents the pressure gradient across the valve that would push the valve close to maximum opening. Finding $∆p_{valve}^{t}$ consisted of solving Equation (7) for $∆p_{valve}=∆p_{valve}^{t}$ with $R_{valve}^{m}=1.05R_{vl}$. This led to the following expression:

(11) $$∆p_{valve}^{t}=\frac{1}{s}ln\left(\frac{R_{vh}}{0.05R_{vl}}\right)$$

### 2.4. Analysis

All the calculations were done in Python programming language by developing a Jupyter notebook [38]. Data were stored and manipulated with the Pandas library of Python [39]. Basic mathematical operations were done with the NumPy library [40]. The linear fitting to estimate $R_{conn}$ was achieved with the “linregress” function of the Scipy library [41]. All other model fittings were achieved with the “minimize” function from the LMFIT library [42] with the Nelder–Mead numerical minimization method [43]. All of the plots were realized with the Matplotlib library (v3.10.0) [44].

The coefficient of determination $R^{2}\in \left[0,\;1\right]$ was used as a measure of the correlation between model-derived values and experimental data. It is defined as:

(12) $$R^{2}\left(B\right)=1-\frac{\sum_{q=1}^{n}{\left(B_{q}^{m}-B_{q}^{exp}\right)}^{2}}{\sum_{q=1}^{n}{\left(B_{avg}^{exp}-B_{q}^{exp}\right)}^{2}}$$

where B refers to the quantity being modeled, the superscript m and exp indicates model estimated and experimental values of B, respectively. The subscript “avg” indicates the average of the experimental values over all n data points. A high value of $R^{2}$ indicates that a good fit is globally obtained. Typically, $R^{2}>0.9$ is sought after and this is what we aimed for.

## 3. Results

### 3.1. Bench Measurements

**Figure 3:**
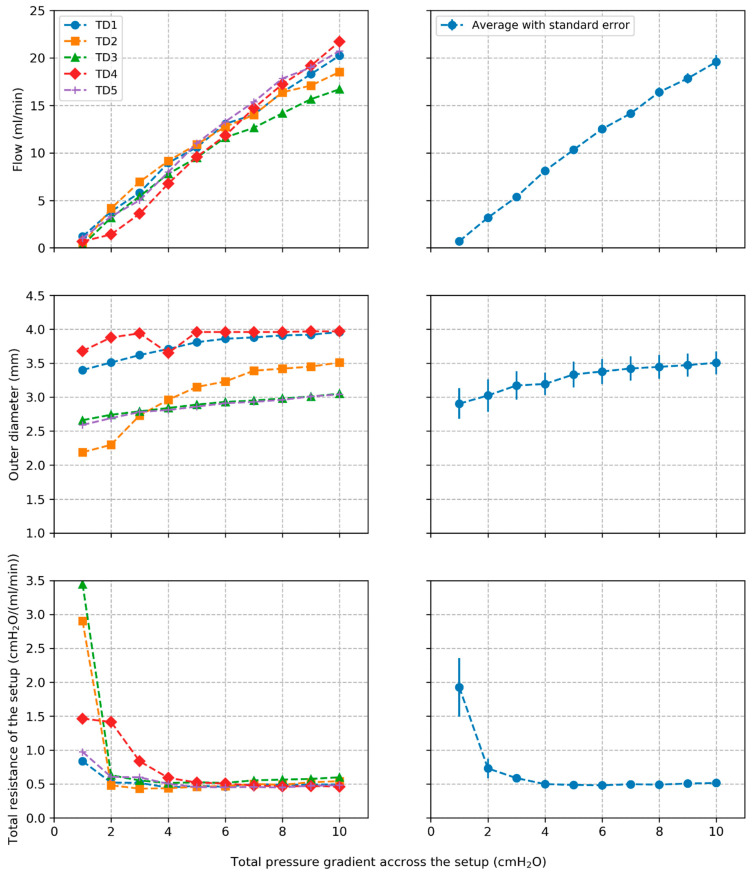
Plots of volumetric flow rate, outer diameter, and total resistance of the setup along with average values and standard errors across all tested segments.

Plots of volumetric flow rate Q across the setup and outer diameter $D_{out}$ of the TD segments against pressure gradient across the setup ${∆p}_{total}$ are shown in Figure 3 for the individual segments along with average behavior and standard errors over all segments. The total resistance $R_{total}$ of the setup is also shown for the different TD segments. The wall thickness was 0.11+/−0.04 mm, which is consistent with our previous study [29].

### 3.2. Resistance of the Connectors

**Figure 4:**
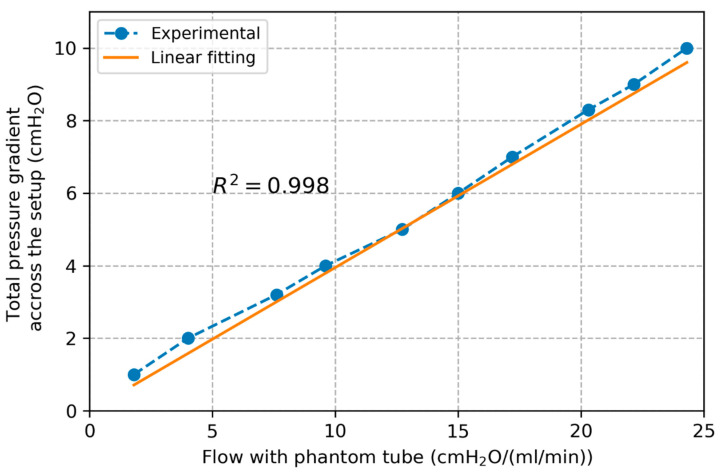
Plot of pressure gradient across the setup and flow rate with the phantom tube along with linear interpolation line.

Plots of volumetric flow rate against pressure gradient across the setup are presented in Figure 4 for the test with phantom tube. The pressure–flow relation was verified to be linear as assumed ($R^{2}=0.992$ for the linear correlation) with a slope of 0.395 cmH_2_O/(mL/min) corresponding to the total resistance of this setup. From Equation (4) we found $R_{phantom}=1.86\times 10^{-3}$ cmH_2_O/(mL/min). Finally, $R_{phantom}$ was subtracted from the total resistance of the system to return the value of $R_{conn}=0.393$ cmH_2_O/(mL/min).

### 3.3. Resistance of TD Vessel and Valve

**Figure 5:**
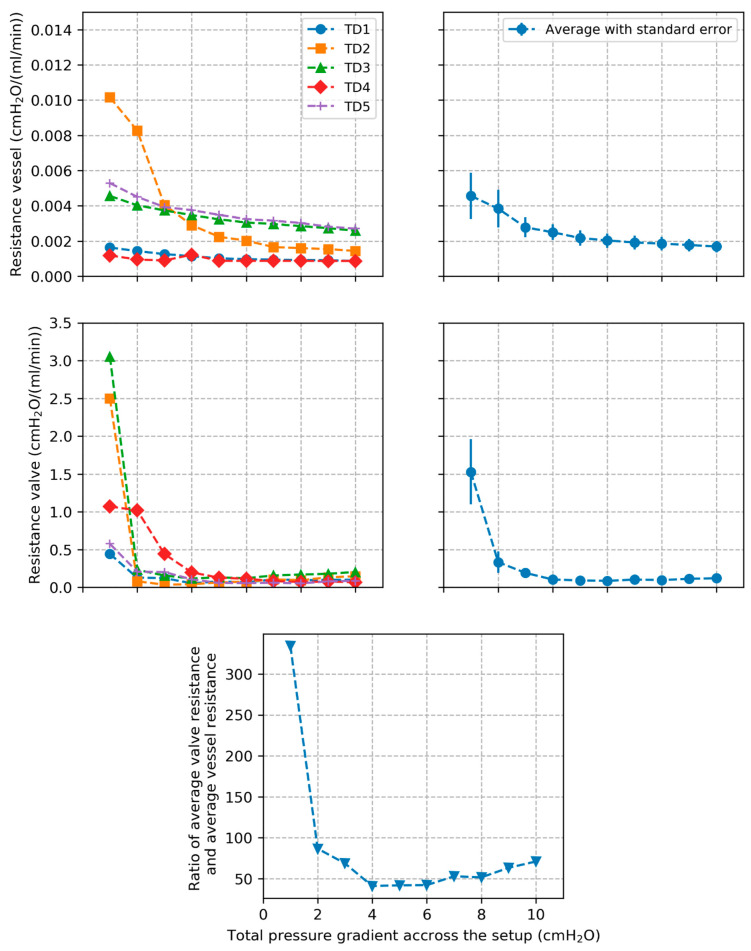
Plots of TD vessel and valve resistances along with average values and standard errors across all tested segments. Plot of the ratio between average valve resistance and average vessel resistance across all TD segments is also presented.

Plots of vessel resistance $R_{vessel}$ and valve resistance $R_{valve}$ estimated from Equations (5) and (6), respectively, are shown in Figure 5 for the individual segments along with average behavior and standard errors. Evolution of the average $R_{valve}$/$R_{vessel}$ ratio over all TD segments is also presented.

### 3.4. Model of TD Valve Resistance

**Figure 6:**
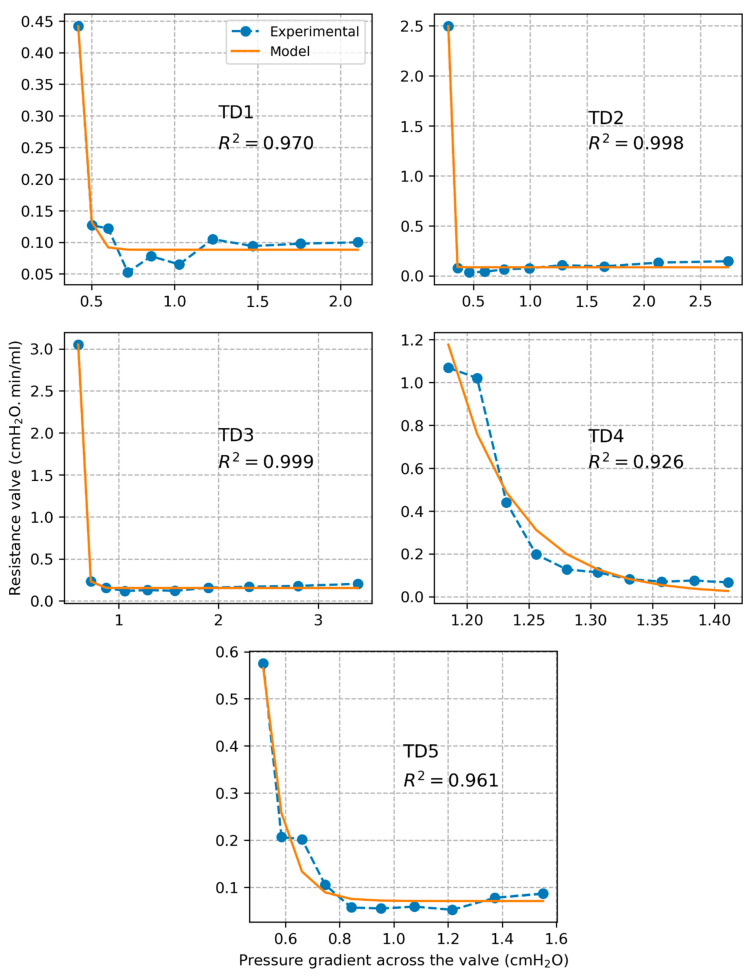
Plots of TD valve resistance along with estimations with the fitted sigmoidal model.

**Figure 7:**
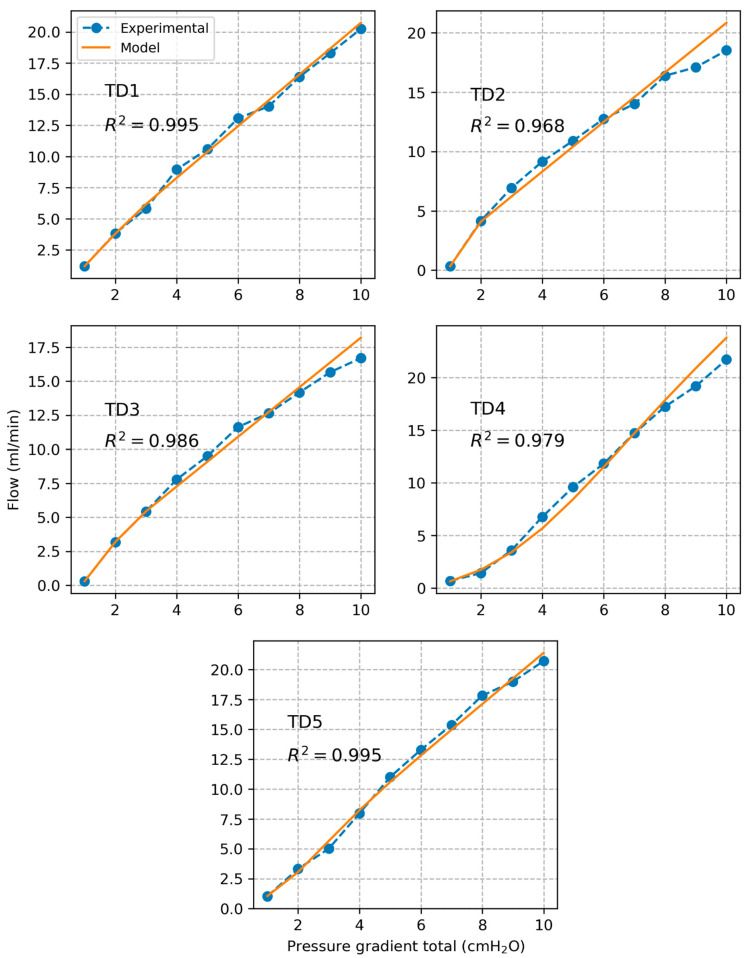
Plots of measured volumetric flow rate along with prediction based on the model of the valve resistance.

Plots of experimentally calculated valve resistance $R_{valve}$ and corresponding model estimated values $R_{valve}^{m}$ are presented in Figure 6 for each individual segment as a function of pressure gradient across the valve ${∆p}_{valve}$. The correlation coefficient between experimental and model-based valve resistance values was found to be $R^{2}=0.971\pm 0.027$. The estimated model parameters are listed in Table 1. Based on the model, we calculated that a plateau valve resistance value is reached for an average threshold pressure gradient value of $∆p_{valve}^{t}=0.84\pm 0.42\;$cmH_2_O over all tested segments. Plots of experimentally measured volumetric flow rate and corresponding values estimated based on the valve resistance model are shown in Figure 7. The correlation coefficient between experimental and model-based flow values was found to be $R^{2}=0.985\pm 0.010$.

## 4. Discussion

To our knowledge, only two previous studies have evaluated the resistance to flow of the TD. Both assumed a constant resistance value although it is bound to change due to changes in vessel diameter and valve opening with variation of pressure gradient [25,26]. This is the first study investigating the pressure–flow relation in the TD accounting for individual vessel and valve resistances. TD was dissected from five domestic swine and segments with one valve were tested using a special apparatus to characterize the pressure–flow relation of the segments in the passive state. Through a mathematical analysis of the results, the resistances of porcine TD vessel and valve were characterized. Poiseuille’s law was used to model the vessel resistance and a sigmoidal function was found to represent well the resistance to flow of the valve.

### 4.1. TD Vessel and Valve Resistances

TD vessel resistance decreased rapidly for all tested segments as pressure gradient increased and then converged towards a constant value. This correlated with the pressure–diameter behavior of the TD vessel which showed the inverse trend. The trend highlights that the TD becomes rapidly stiffer as pressure increases. The TD valve resistance shows a similar behavior consisting of a rapid decrease followed by convergence towards a constant value. This suggests that the TD valve opens rapidly as the pressure gradient across the valve increases, leading to a decrease of the resistance to flow, then reaches maximal opening resulting in a constant value of the resistance. The resistance of the TD valve was on average one to two orders of magnitude higher than vessel resistance which suggests that flow resistance in the TD is mainly due to the valve.

### 4.2. Model of the Valve Resistance

The sigmoidal model of valve resistance proposed in previous studies of lymphatic vessels captured very well the behavior of the TD valve ($R^{2}=0.971\pm 0.027$). The model was validated by comparing predicted flow values to measured flow values $(R^{2}=0.985\pm 0.010$). Based on the model, we estimated that the valve opens close to its maximal capacity at a pressure gradient of about 0.84 cmH_2_O.

### 4.3. Limitations

The current bench setup to measure flow rate only recorded constant passive flow through the TD segment. Therefore, the proposed model is purely static and does not account for dynamic effects. In future tests, periodic inlet flow should be introduced to measure the dynamic resistance of the valve. The application of external pressure on the lymphatic vessel develops a pressure gradient in the TD which enables lymph flow to drain into the venous system. Future studies should also include external loading and measure its impact on the pressure gradient and the flow rate through the system. Additionally, our current setup did not consider the resistance offered by the valve due to retrograde flow or adverse pressure gradient, which should be investigated in future studies. Because TD walls are thin and soft, the change of both diameter and flow were large when pressure increased from 0 to 1 cmH_2_O, which also resulted in a large magnitude of variation. The dissections could have damaged the integration of TD. For instance, the partial wall of the TD segment may have been randomly excised, which may be a cause for the large variation in the measurement across segments. Finally, the proposed model of the valve resistance is sensitive to pressure variation in the low-pressure regime due to its exponential nature, which could impact the estimation of the model parameters $R_{vh}$ and s. Future improvement could include experimental setups that allow for more granular measurements in the low-pressure range to obtain more data points and then more accurate estimation of the parameters through better model fitting.

### 4.4. Implications of the Findings

There have been very few studies conducted on large animal TD, which hinders translational advancements in lymphatic research. Moreover, most of the studies on large animals focused on sheep [10,45] and dogs [46]. Although there are similarities in the anatomy and physiology between porcine and humans, the data on the flow behavior of the swine TD is unavailable. Here, we established passive relationships for TD vessel and valve resistances. These relations and associated model parameters provide crucial components to model the pumping of lymph in swine TD. Once the remaining components (listed in the previous paragraph) are established, the resulting full TD flow model could help better understand parameters affecting TD flow in health and disease. Use of more advanced modeling techniques [47,48] can be considered as well to guide the design of more efficient approaches for thoracic duct decompression.

## 5. Conclusions

We established a model that captures the relation between resistance to flow, pressure gradient, and diameter in a TD segment with one valve under passive condition. The model was validated against experimental data in a translational animal model. To our knowledge, this is the first such validated model. It provides the foundation for developing models that accurately simulate lymphatic flow in the entire TD. Such models are essential for enhancing our understanding of lymphatic flow in the TD, which plays a crucial role in many conditions such as peripheral edema, ascites, and hepatomegaly. Future research could focus on establishing such models, as well as studying the resistance to flow under active conditions.

## Figures and Tables

**Table 1:** Estimated parameters of the valve model for all the TD segments tested.

TD	Rvl (cmH_2_0/(mL/min))	Rvh (cmH_2_0/(mL/min))	s (cmH_2_0^−1^)
1	0.088	1.54 × 10^4^	25.4
2	0.085	6.42 × 10^10^	85.8
3	0.154	6.33 × 10^7^	28.5
4	0.011	7.38 × 10^9^	19
5	0.071	9.09 × 10^2^	14.5

## Data Availability

The data generated in this paper correspond to the bench experiments measurements. It is compiled in a “bench_data.xlsx” file that is openly available on Zenodo under the under the permissible Creative Commons Attribution 4.0 International (CC-BY) license [49]. The code associated with this manuscript consists of a “main.ipynb” Jupyter notebook that contains the code used to analyze the data and generate the results. It is shared under the permissible MIT license on Zenodo [50]. The notebook was made FAIR (Findable, Accessible, Interoperable, Reusable) according to the FAIR-BioRS guidelines [51].
